# COVID-19 Pandemic as a Driver for Spreading Virtual Care Globally: The Future Starts Now

**DOI:** 10.6061/clinics/2020/e1967

**Published:** 2020-05-18

**Authors:** Rafael Denadai

**Affiliations:** I Instituto de Cirurgia Plastica Cranio Facial, Hospital SOBRAPAR, Campinas, SP, BR.

As the COVID-19 pandemic spreads, healthcare systems are crashing worldwide. Public health authorities are focusing their decision-making processes toward best allocation of resources and personnel for the development of vaccines, establishment of effective treatments, management of affected patients, and protection of citizens and the workforce from human-to-human infection (1). Further, the healthcare workforce should act in alignment with the quickly evolving evidence (2). In this unparalleled healthcare scenario, a pertinent question has arisen: How do clinicians provide the best care for patients with diseases other than COVID-19-related diseases (3)? The Brazilian Federal Council of Medicine has authorized teleconsultation during the ongoing pandemic (4). I believe that timely implementation of virtual consultations can not only promote care provision for these patients in private and public healthcare sectors but also help generate a database that can transform healthcare systems.

The key advantages of telemedicine is its mobility (5), and it presents a perfect match for the current public health policy of social distancing and cancellation of non-urgent consultations and surgeries. In light of the current pandemic, countries that under normal circumstances do not permit virtual clinician-patient consultation for virtual care are now supporting the widespread use of telemedicine. This call for virtual technology-assisted care provision exposes a historically wide range of telemedicine-related issues, including legal issues and issues with funding; guidelines; and different degrees of experience in telehealth among countries, hospitals, and clinicians (6,7). The differences in regulations and practices may produce uncertainty regarding licensure, data privacy, reimbursement, confidentiality, patient consent, and accessibility that could delay clinicians from adopting telehealth practices. Previously described telemedicine data (5-7) aligned with the day-to-day rise in COVID-19-based clinical recommendations (2) could aid clinicians in judiciously redirecting their regular practices toward virtual-based care.

Noticeably, this global crisis, which is marked by uncertainty, could also create a golden opportunity to implement structured workflows for building a virtual consultation-focused database. Clinicians should now recognize that delivering “COVID-19-adjusted virtual care,” aligned toward gathering reliable data, needs to be a priority to support a change toward the delivery of “lessons learned care” and “data-driven virtual care” globally. Data collected at the point-of-care at a provider level could comprise a large key dataset (8) that can be applied in future telemedicine-focused regulation policy at the government level.

Once this catastrophic COVID-19 outbreak has been overcome, the standard of care may change compared to the pre-pandemic standard, and I expect that the barriers restraining the widespread use of telemedicine (6,7) could also be overcome, or at least a pathway to achieve this goal may be demarcated. In a post-COVID-19 era, a possible change in healthcare could see “in-person health care as option B” (5). The future starts now, and clinicians should act accordingly.
